# The (Poly)phenol-Carbohydrate Combination for Diabetes: Where Do We Stand?

**DOI:** 10.3390/nu15040996

**Published:** 2023-02-16

**Authors:** Ana Marta de Matos, Regina Menezes

**Affiliations:** 1 Centro de Química Estrutural, Institute of Molecular Sciences, Departamento de Química e Bioquímica, Faculdade de Ciências da Universidade de Lisboa, Campo Grande, 1749-016 Lisboa, Portugal; 2 CBIOS—Universidade Lusófona’s Research Center for Biosciences & Health Technologies, Campo Grande 376, 1749-024 Lisboa, Portugal; 3 iNOVA4Health, NOVA Medical School|Faculdade de Ciências Médicas, NMS|FCM, Universidade Nova de Lisboa, Campo dos Mártires da Pátria 130, 1169-056 Lisboa, Portugal

**Keywords:** Pan-Assay Interference Compounds, invalid metabolic panaceas, functional nutrients, natural product-based drug discovery, *C*-glucosylation

## Abstract

The type 2 diabetes epidemic is real and hardly coming to an end in the upcoming years. The efforts of the scientific community to develop safer and more effective compounds for type 2 diabetes based on the structure of natural (poly)phenols are remarkable and have indeed proven worthwhile after the introduction of gliflozins in clinical practice. However, low-quality reports on the antidiabetic potential of plant-derived lipophilic (poly)phenols continue to pile up in the literature. Many of these compounds continue to be published as promising functional nutrients and antidiabetic pharmaceutical leads without consideration of their Pan-Assay Interference Compounds (PAINS) profile. This evidence-based opinion article conveys the authors’ perspectives on the natural (poly)phenol artillery as a valuable and reliable source of bioactive compounds for diabetes. Ultimately, in light of the already established membrane-perturbing behavior of lipophilic (poly)phenols, together with the multiple benefits that may come with the introduction of a *C*-glucosyl moiety in bioactive compounds, we aim to raise awareness of the importance of contemplating the shift to (poly)phenol–carbohydrate combinations in the development of functional nutrients, as well as in the early stages of antidiabetic drug discovery.

According to the International Diabetes Federation (IDF), 537 million adults are presently living with diabetes, which corresponds to a global prevalence of 10.5% [1]. If the trend evolves as predicted, the number of adults with diabetes will have risen to an astonishing 783 million by 2045 [1]. However, it is estimated that over 90% of the diabetes population has type 2 diabetes—a rather preventable and, at times, even reversible type of diabetes that mostly arises from unhealthy lifestyle choices, including the consumption of foods with a high fat content and lack of physical activity [1,2]. Dietary changes towards an increased intake of nutritious and bioactive-rich plant-based foods, such as whole grains, nuts, seeds, legumes, fruits, and vegetables, together with reduced consumption of saturated fatty acids, refined sugars, and red meats, have been proven to work effectively in the prevention and management of type 2 diabetes [3,4]. As much as these changes in diet are encouraged with a primary focus on weight loss and (poly)phenol-mediated gut microbiota modulation to achieve physiological insulin sensitivity and normal pancreatic function [4,5,6], the exploitation of the most recommended foods as natural sources of antidiabetic compounds is not only logical but also undeniably tempting for scientific community members looking for functional nutrients, bioactives, and lead compounds in antidiabetic drug discovery.

From in vitro to clinical studies, the number of published reports covering the potential of dietary lipophilic (poly)phenols to treat diabetes and its comorbidities is countless [7,8]. Resveratrol, curcumin, genistein, quercetin, phloretin, caffeic acid, and epigallocatechin gallate—just to name a few—are remarkably popular natural compounds that, together with their glycosides, make up some of the most vastly studied sets of molecules for diabetes. In spite of the high amount of in vitro cell-based assays claiming promising (poly)phenol-induced antidiabetic effects, those results translate very poorly into successful outcomes in clinical studies [9]. Indeed, the antidiabetic effects of natural (poly)phenols in human subjects are often small or non-significant, with high variability between studies [9]. Overall, the existing data has been found to be rather limited and even contradictory, highly contrasting with lab bench research. Study design quality, population heterogeneity, interindividual variability, low compound bioavailability, and administration of multi-component mixtures (e.g., plant extracts) are some of the reasons frequently pointed out to explain these contradictions. Moreover, even when statistically significant antidiabetic effects are found, studies often fail to prove that these are sustainable in time with no relevant toxic effects [9]. All of these complex issues call for internationally harmonized natural product research recommendations. However, the fact that many of the target polyphenols are nowadays well-known for their promiscuous behavior is often forgotten.

In the context of medicinal chemistry applied to drug discovery, Pan-Assay Interference Compounds (PAINS) were described for the first time in 2010 as molecules with structural features that are able to interfere with drug screening assays, often leading to false positive results [10]. These features can account for their effects as redox cyclers, metal chelators, covalent modifiers, self-aggregators, and even membrane disruptors which, in turn, prevent PAINS from having their structure rationally optimized into potential drug candidates with adequate target selectivity and low toxicity [10,11]. This concept can be applied not only to synthetically made compounds but also to molecules derived from natural sources—the so-called invalid metabolic panaceas (IMPS) [12,13]. In particular, plant-derived lipophilic compounds with several hydroxy groups, such as curcumin, epigallocatechin gallate, quercetin, genistein, and resveratrol have been put in the spotlight for their action as membrane disruptors, bearing the capacity to modulate the activity of membrane receptors and transmembrane proteins [10]. This capacity arises from a fine combination of lipophilicity, molecular planarity, and the presence of multiple hydroxy groups, which allows the compounds to be inserted into the cell membrane and consequently modify their physiological properties [14]. Phloretin (Figure 1), for instance, has been known for its ability to reduce membrane dipole potential since 2013 [15]. Still, it was in 2021, with the combined study of resveratrol, genistein, and phloretin, that membrane dipole potential reduction was proposed as a mechanism by which lipophilic polyphenol PAINS or IMPS are able to interfere with the electric profile of membranes in a non-specific way [14]. Strikingly enough, the *C*-glucoside of phloretin, nothofagin (but not the *O*-glucoside, phlorizin), did not follow the aglycone in its promiscuous behavior. These results were impeccably corroborated by *C*-glucosyl resveratrol and *C*-glucosyl genistein—so much so that *C*-glucosylation was proposed as a tool to prevent PAINS-induced membrane dipole potential alterations [14]. Here, it is important to understand that membrane dipole potential changes are strongly implicated in the regulation of transmembrane protein conformation and function, particularly in cholesterol-enriched domains such as lipid rafts [16]. As changes in protein activity may have a profound effect on the way intracellular signaling cascades are activated or inhibited, compounds that are able to alter the membrane dipole potential may therefore elicit illusory pharmacological effects that are not caused by any specific drug–target interaction.

Although the exact mechanisms by which *C*-glucosyl (poly)phenols are able to prevent membrane dipole potential alterations caused by (poly)phenol PAINS are unknown, there are some theories. As highlighted elsewhere [14], these compounds are expected to locate more superficially in the membrane compared to their aglycones, which may completely change the way (poly)phenol hydroxy groups remodel the naturally existing hydrogen-bond networks between phospholipid headgroups and surface water molecules, as well as the relative orientation of their electric dipoles. As the membrane dipole potential is strongly affected by the way water molecules are positioned on the surface of the membrane, the modulation of surface hydration mechanisms can, indeed, result in significant membrane dipole potential alterations [14]. Furthermore, it is not yet entirely understood why *C*-glucosyl polyphenols are so unique in achieving this effect when compared to their *O*-glucosyl counterparts. Nonetheless, fewer rotatable bonds arising from the replacement of the anomeric C-O-C triad by a C-C bond is expected to result in enhanced molecular rigidity, which ought to more favorably sustain the conformation of the molecule that promotes proper membrane insertion for minimal changes to the membrane dipole potential.

More extensive experiments are warranted to confirm the hypothesis that *C*-glucosylation can in fact be used as a general way of converting membrane-disrupting lipophilic polyphenols into non-IMP (poly)phenols for a wider range of molecular entities. In this context, however, it is also important to reinforce that such a strategy would only be valid for (poly)phenols that do not check the box for other types of PAINS/IMPS features, and many of them actually do. Catechol and hydroquinone moieties, which are very frequently found in natural products, are both prone to autoxidation followed by covalent modification, likely leading to the formation of multiple and non-specific protein adducts often masked as protein aggregation inhibition [19,20,21]. This does not mean that compounds with these features should be immediately discarded, but rather that appropriate assays must be conducted in order to exclude this (quite probable) possibility before any claims of groundbreaking bioactivity are established.

Notwithstanding, for natural (poly)phenols that do pass that stage, there are strong hints that *C*-glucosylation should be considered when it comes to the pursuit of non-reactive lipophilic (poly)phenols with antidiabetic properties: (1) *C*-glucosides extracted from natural plant sources are increasingly recognized as functional ingredients for their potent antihyperglycaemic, anti-aggregation, and antioxidant bioactivities, both alone or in combination with other antidiabetic compounds [17,22,23,24,25,26]; (2) sugar-linked compounds may exhibit improved toxicity, solubility, and membrane permeability profiles versus their aglycones, which may enhance their bioavailability [27,28,29]; (3) when compared to *O*-glycosides, their (poly)phenol *C*-glucosyl analogs may also lead to improved selectivity and metabolic stability [17,30]; (4) as mentioned above, they seem to prevent membrane-disrupting effects of lipophilic (poly)phenol PAINS/IMPs [14]. Let us consider the successful case of gliflozins, a class of *C*-glucosyl aromatic compounds originally developed and approved as SGLT2 inhibitors for the treatment of type 2 diabetes [31]. The primary ancestor of gliflozins is the natural membrane dipole potential reducer phloretin, found in apples, pears, and other fruits [32]. This low-affinity and non-selective sodium-glucose cotransporter 2 (SGLT2) inhibitor (SGLT2 IC_50_ 25,000 nM vs. SGLT1 IC_50_ 50,000 nM [18]) sees its potency enhanced with the introduction of an *O*-glucosyl moiety (SGLT2 IC_50_ 21–67 nM vs. SGLT1 IC_50_ 290–499 nM [22,32]), but it is not until it is linked to a *C*-glucosyl group that both potency and selectivity are dramatically improved (SGLT2 IC_50_ 12 nM vs. SGLT1 IC_50_ 19,000 nM [17]) (Figure 1). Notably, selective SGLT2 inhibition is crucial in avoiding gastrointestinal adverse events [33]. Even though Washburn and colleagues were inspired by natural *O*-glucoside for the development of the first SGLT2 inhibitor to receive regulatory approval—dapagliflozin (SGLT2 IC_50_ of 1.1 nM vs. SGLT1 IC_50_ 1400 nM [32])—the team quickly realized that *C*-glucosylation was the only way to prevent extensive in vivo sugar–aglycone hydrolysis [33]. This and several other steps of structural optimization culminated in the approval of dapagliflozin in 2014, a life-changer for millions of diabetic patients around the world. Many more gliflozins have been developed for the treatment of type 2 diabetes since, and additional indications focused on typical comorbidities of diabetes, such as heart and kidney disease, have been approved in recent years [31]. This further highlights the intrinsic value of these *C*-glucosyl aromatic molecules as multitarget antidiabetic agents.

All in all, gliflozins serve as a great illustrative example of the power of *C*-glucosylation when considering the development of (poly)phenol-based functional nutrients and drug-like molecules. We recommend testing natural (poly)phenols regarding membrane interference prior to being published for their promising bioactivity in cell-based assays (Figure 2), hence counteracting the presumption that they owe their broad antidiabetic effects to specific interactions with biological targets. We also encourage consideration of *C*-glucosylation as a means of converting membrane-disrupting IMPS into compounds that, at the very least, are much more likely to progress to the market as antidiabetic pharmaceuticals or functional nutrients. It is important to note that, although more technically challenging than *O*-glucosylation, (poly)phenol *C*-glucosylation is entirely feasible both synthetically and through biotransformation, with an increasing number of methodologies being created and made available to the scientific community [34,35,36].

Finally, it should also be made clear that while the rationale herein proposed focuses on the development of small compounds with robust antidiabetic activity against specific molecular targets for the treatment and management of type 2 diabetes, this does not imply that dietary non-glycosylated lipophilic (poly)phenols cannot have an important adjuvant role with regard to the prevention of this condition. In fact, lipophilic (poly)phenols have been shown to positively modulate the composition of gut microbiota, including the above-described IMPS resveratrol and curcumin [6,37].With mounting evidence supporting the link between gut microbiota dysbiosis and the development of obesity, insulin resistance, and glucose intolerance, among other metabolic dysfunctions, beneficial changes in gut microbiota composition alone may predispose the host towards a lower risk for metabolic syndrome [38,39].Therefore, this opinion article is not intended to discourage the regular consumption of naturally (poly)phenol-enriched plant-based foods in any way; rather, we aim to encourage natural product and medicinal chemists to carefully evaluate the validity of any (poly)phenol bioactivity claims, while working to find solutions for the frequently encountered problems of lipophilic (poly)phenol IMPS.

In summary, given their potential for improved bioactivity and non-PAINS profile regarding membrane disruption, polyphenol *C*-glucosides deserve more credit than they have been given as lead molecules for both the development of functional food ingredients and pharmaceuticals for type 2 diabetes. Aromatic *C*-glucosylation is feasible through a variety of methods, and has not only been proven to enable the fine-tuning of (poly)phenol antidiabetic effects and drug-like properties, but has also led to tangible results when it comes to the discovery of nature-inspired molecules able to make a difference in the treatment of type 2 diabetes and its comorbidities, as exemplified with the case of gliflozins. There is no question that natural (poly)phenols are valuable sources of active scaffolds; the question is simply about understanding how to use them and, above all, how to make them thrive.

## Figures and Tables

**Figure 1:**
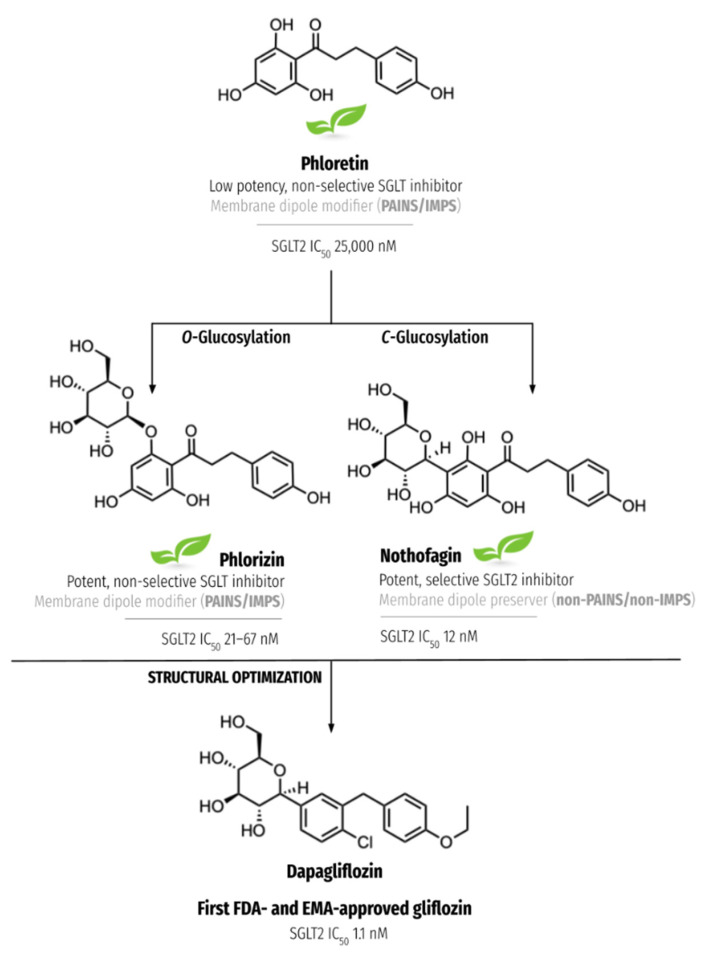
The natural structural heritage of dapagliflozin: Effects of *O-* and *C*-glucosylation in the potency, selectivity, and membrane-disrupting PAINS/IMPS profile of the natural lipophilic (poly)phenol phloretin. The green leaf icon indicates the presence of the compound in natural sources. IC_50_ values were retrieved from refs. [17,18].

**Figure 2:**
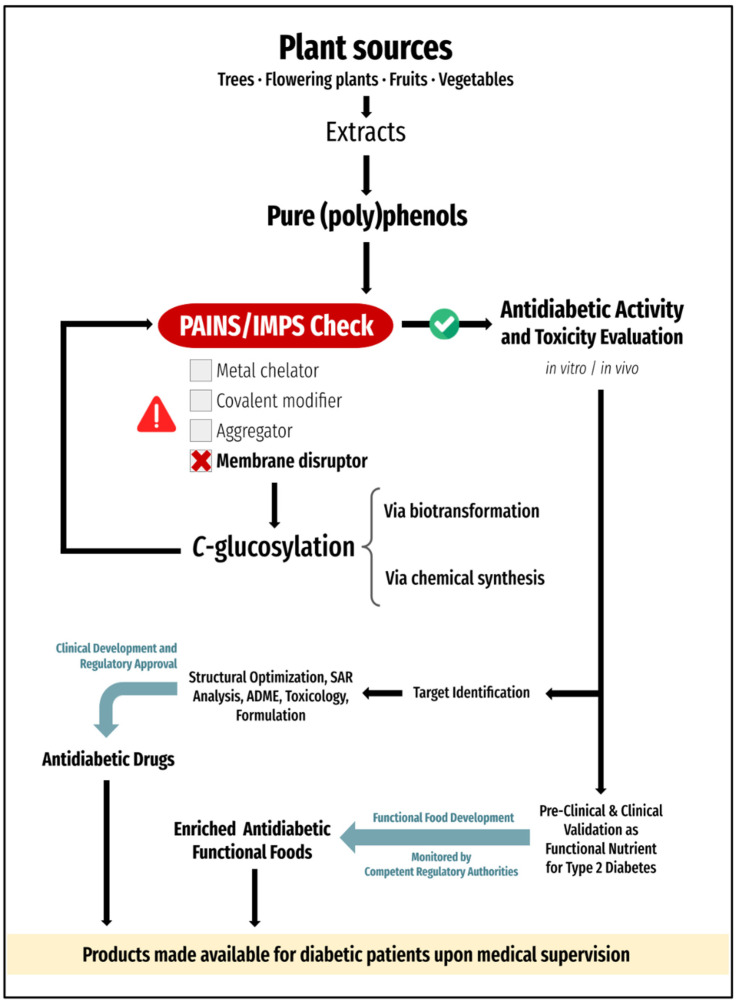
Proposed stepwise approach for the identification/development of natural (poly)phenol-based compounds for type 2 diabetes, either as functional nutrients or as potential pharmaceutical agents. All compounds should be purified and carefully examined regarding their potential PAINS/IMPS-type behavior. In the case of non-glycosylated planar lipophilic (poly)phenols, which bear an intrinsic risk for behaving as membrane disruptors, *C*-glucosylation may be considered, either via biotransformation or chemical synthesis, in an attempt to halt non-specific interactions with cell membranes. Non-PAINS/non-IMPS can then be evaluated regarding their antidiabetic bioactivity and toxicity, both in vitro and in vivo. Compounds being assessed and/or developed as functional nutrients for type 2 diabetes should be validated as such in both pre-clinical and clinical studies, which must include the evaluation of long-term antidiabetic and toxic effects. With a favorable benefit/risk profile, such functional nutrients could be incorporated into enriched antidiabetic functional foods, monitored by competent regulatory authorities, and recommended to patients under medical supervision. For drug discovery and development, target identification is mandatory (i.e., should be conducted if not yet established in the first bioactivity experiments), after which structural optimization and pharmaceutical development can potentially lead to regulatory approval and launch.
